# Identification of Novel Type Three Secretion System (T3SS) Inhibitors by Computational Methods and Anti-*Salmonella* Evaluations

**DOI:** 10.3389/fphar.2021.764191

**Published:** 2021-11-16

**Authors:** Yonghui Wang, Meihui Hou, Zhaodong Kan, Guanghui Zhang, Yunxia Li, Lei Zhou, Changfa Wang

**Affiliations:** ^1^ College of Agronomy, Liaocheng University, Liaocheng, China; ^2^ Burns and Plastic Surgery Department, The 960th Hospital of the PLA Joint Logistics Support Force, Jinan, China; ^3^ Laizhou City Laiyu Chemical Co., Ltd., Laizhou, China; ^4^ School of Biological Science and Technology, University of Jinan, Jinan, China

**Keywords:** type III secretion system inhibitor, virtual screening, molecular docking, *Salmonella* species, anti-bacterial activity

## Abstract

Three type III secretion system (T3SS) inhibitors (compounds 5, 19, and 32) were identified by virtual screening and biological evaluation. These three compounds were evaluated against a panel of *Salmonella* species strains including *S. enteritidis*, *S. typhi*, *S. typhimurium*, *S. paratyphi*, and *S. abortus equi*, and their minimum inhibitory concentrations ranged from 1 to 53 μg/ml. Especially, these compounds showed comparable activity as the of the positive control gatifloxacin towards *S. abortus equi*. The present results suggest that these new T3SS inhibitors could be used as a potential lead molecule for drug development of anti-*Salmonella*.

## Introduction

Salmonellosis is a general term for infectious diseases of livestock and poultry caused by *Salmonella* bacteria. *Salmonella*, belonging to the *Enterobacteriaceae* family, are foodborne zoonotic enteric and facultative intracellular pathogenic bacteria, which are one of the most common causes of intestinal infections worldwide (Akiba et al., 2011). *Salmonella* are mainly categorized into two species including *Salmonella bongori* and *Salmonella enterica*, especially the last of which has a large number of subspecies and serovars, and some of them, such as serovar *Enteritidis*, are highly pathogenic (Vullo et al., 2011). It mainly causes abortion or death of pregnant female animals, such as pigs, chickens, cattle, donkey and other animals, with the symptoms of fever, diarrhea, sepsis, gastroenteritis and meningitis, as well as intestinal damage in both humans and animals (Zha et al., 2019). The extensive resistance of *Salmonella* to a variety of antibiotics (ampicillin, chloramphenicol, streptomycin, antimicrobial sulfonamides and tetracycline) has been reported worldwide (Arcangioli et al., 2000; Mąka et al., 2015; Tran-Dien et al., 2018; Vilela et al., 2019; Mengistu et al., 2020). Thus there is an urgent need for the discovery of new anti-*Salmonella* drugs.

The type III secretion system (T3SS) is a nanomachinery protein utilized by many Gram-negative bacteria to inject bacterial effector proteins from the bacterial cytoplasm directly into the eukaryotic host cells. Although the detailed molecular mechanisms of the bacterial effector functions and the biogenesis of T3SSs are still need extensive research, biochemical and genetic studies of lots of Gram-negative bacterial pathogens have indicated that effector translocation by T3SSs is vital for successful infection (Galan, 2001). T3SS is required to initiate infections and central to the virulence of many pathogenic bacteria, such as *Salmonella* and *Shigella* (Tsou, et al., 2013). The mainly structure of T3SS is a needle apparatus composed of more than 20 different proteins (Cornelis, 2006), of which the tip complex are essential component consisted of tip proteins and translocon proteins, and tip proteins bound on the top of the needle, while the translocon proteins are membrane-spanning proteins. The T3SS tip proteins are SipD (Lara-Tejero and Galan, 2009) in the *Salmonella typhimurium* pathogenicity island 1 (SPI-1). T3SS has been regarded as promising targets for the development of anti-virulence agents, and some T3SS inhibitors have been identified (Hussain et al., 2021). However, there is no T3SS inhibitor reported targeting SipD protein.

**FIGURE 1:**
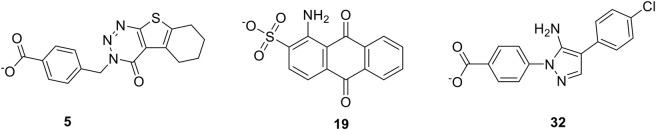
The new T3SS inhibitors presenting in this work.

In this study, the discovery of T3SS inhibitors targeting SipD protein was performed using molecular docking-based virtual screening on Specs database. After the biological evaluation of forty-six purchased compounds that were selected according to the docking screening and structural clustering, three of them, namely, compounds 5, 19, and 32 (Figure 1) with different scaffolds, were found to have effect on the *Salmonella* invasion, which indicated that they were new T3SS inhibitors. The binding model of these compounds with T3SS were investigated and the results indicated that all of them could form tight binding affinity with T3SS tip protein Sip D. Then all these T3SS inhibitors were evaluated for their inhibitory activities towards against a panel of *Salmonella* species strains including *S. enteritidis*, *S. typhi*, *S. typhimurium*, *S. paratyphi*, and *S. abortus equi*. The present study provided new chemotypes for the development of novel T3SS inhibitors targeting SipD protein, which could serve as lead compounds for developing novel medications against *Salmonella* bacteria.

## Results and Discussion

### Molecular Docking Based Virtual Screening Yielded 46 T3SS Inhibitor Candidates

**FIGURE 2:**
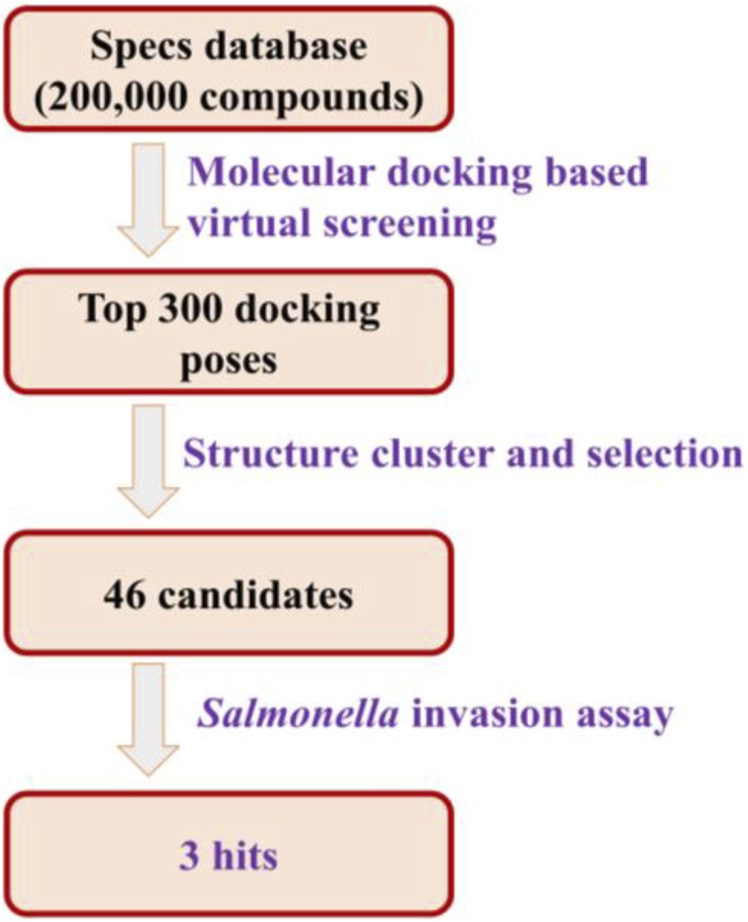
The workflow of the virtual screening.

In order to obtain T3SS inhibitors candidates targeting the tip protein SipD of T3SS, we chose SPECS database to perform molecular docking based virtual screening. The function of SipD protein was documented to promote secretion of effectors and functions at the post-transcriptional and post-translational levels (Glasgow et al., 2017). The needle tip complex in *Salmonella enterica* consists of three translocon proteins: SipB, SipC, and SipD. It is known that knocking out SipD disrupts T3SS regulation to cause constitutive secretion of native proteins (Glasgow et al., 2017). Thus, we used SipD protein to perform virtual screening. According to the docking results, the top 300 docking poses ranked on the docking score were selected for the following cluster analysis and visual selection. Finally, we selected 46 compounds as T3SS inhibitor candidates for the following *Salmonella* invasion assay test. The workflow of the virtual screening was shown in Figure 2. And the detailed method was provided in Supplementary Material.

### The *Salmonella* Invasion Assay Identified three Novel T3SS Inhibitors

**FIGURE 3:**
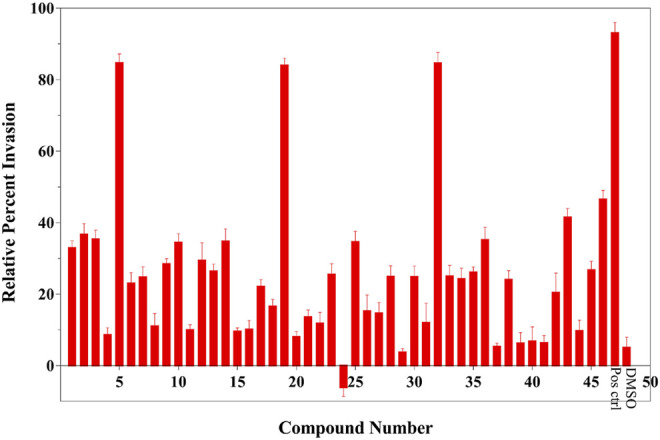
Results of *Salmonella* invasion assay (legends: Pos ctrl: positive control, DMSO: negative control). This result was shown the average of triplicate assay.

To assess the effects of T3SS inhibitor candidates on bacterial invasiveness, we used the *Salmonella* invasion assay to determine the effect of T3SS inhibitor candidates on the ability of *Salmonella* to invade cultured human epithelial cells. Results of *Salmonella* invasion assay with respect to T3SS inhibitors are shown in Figure 3. Overall, most of the 46 compounds could decrease invasiveness, and three of them (5, 19, and 32) showed most drastic effect on the invasiveness.

### Binding Mode Analysis Exhibited Tight Binding Affinity Between 5, 19, and 32 With SipD Protein

**FIGURE 4:**
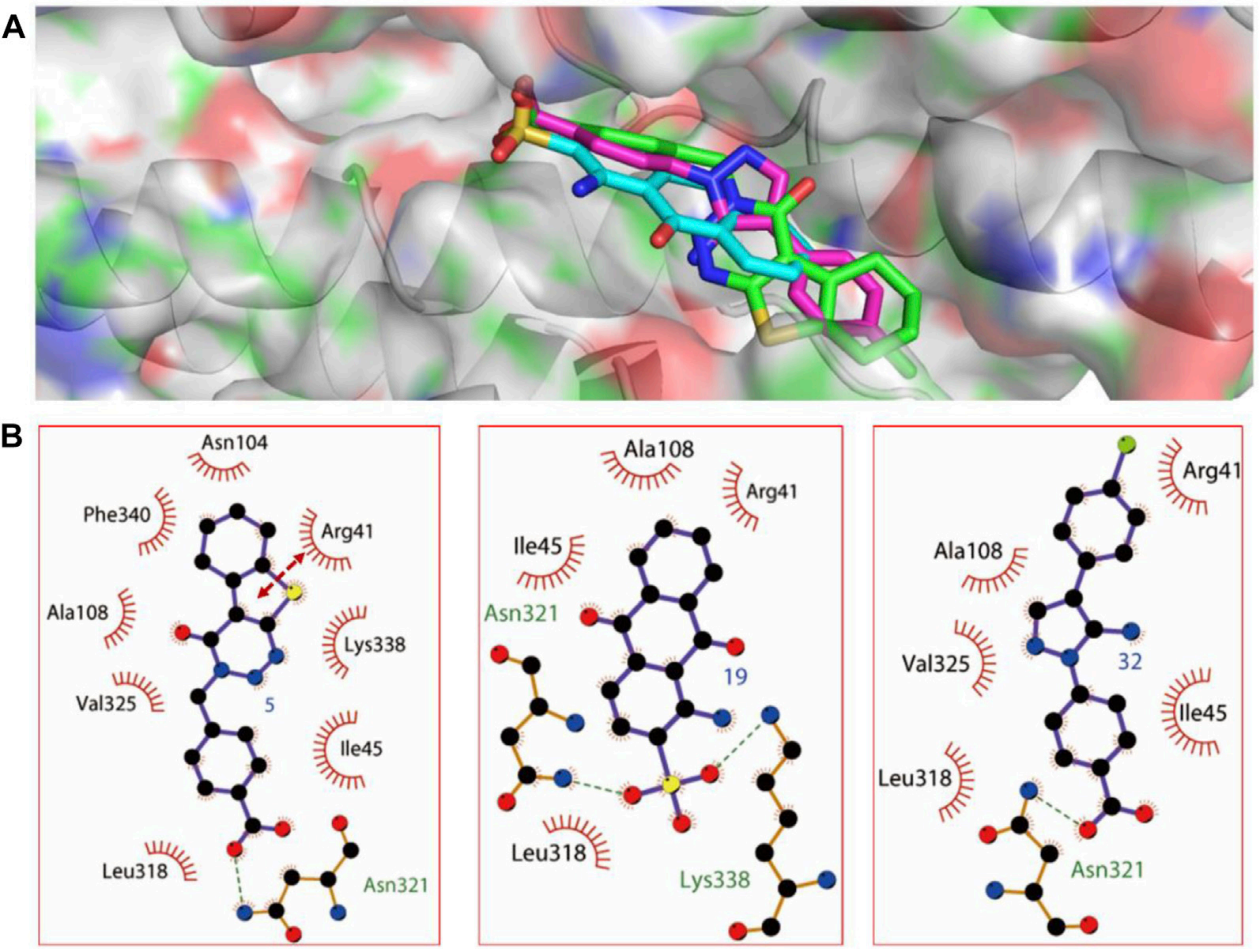
The predicted binding modes of compounds 5, 19, and 32. **(A)** The three dimensional binding mode of compounds 5, 19, and 32. **(B)** The relative schematic diagram showed the hydrophobic interactions (shown as starbursts), H-bond interactions (denoted by dotted green lines), and π-π interactions (displayed as double-head arrow) between compounds 5, 19, and 32 with T3SS tip protein SipD.

The probable binding modes of the three compounds were provided to show the detailed interaction mechanism. As shown in Figure 4, all of the three compounds could occupy the deoxycholate binding site in SipD, with the binding energy showing in Table 1. Compound 5 displayed the most binding interactions than 19, and 32. Compound 5 established hydrophobic interactions with residues Arg41, Ile45, Asn104, Ala108, Leu318, Val325, and Lys338, and formed H-bond and π-π interactions with Asn321 and Arg41, respectively. Compound 19 displayed two H-bond interactions with Asn321 and Lys338, and also established hydrophobic interactions with residues Arg41, Ile45, Ala108, and Leu318. Compound 32 formed H-bond interaction with Asn321, and established hydrophobic interactions with residues Arg41, Ile45, Ala108, Leu318, and Val325. In brief, residues Arg41, Ile45, Ala108, Leu318, and Asn321 were the key residues that showed interactions with all of the three compounds. From the binding mode of the active compounds 5, 19, and 32 with SipD protein, we could see that there was a lot of space in the binding pocket (Supplementary Figure S1) which could be occupied by the inhibitor. Therefore, further structure modification can be performed from the following direction: while maintaining the hydrogen bond interactions, large substituents may be added to the molecular structure of 5, 19, and 32 to increase binding affinity.

**TABLE 1:** Binding energy of the identified compounds.

Compound No	Binding energy (kcal/mol)
5	−10.86
19	−9.45
32	−9.16

### Anti-*Salmonella* Activity Assay of Compounds 5, 19, and 32

The *in vitro* anti*-Salmonella activity* of these found compounds are shown in Table 2. All of them showed potent inhibitory activity against bacteria in *Salmonella* sp., including *S. enteritidis*, *S. typhi*, *S. typhimurium*, *S. paratyphi*, *S. abortus equi*, with MICs ranging from 1 to 53 μg/mL. *S. abortus equi* is well known as the aetiological agent of equus abortion, and interestingly, almost all of these compounds exhibited the most sensitive activity towards *S. abortus equi*, with MICs ranging from 1 to 8 μg/ml, almost as similar as that of positive drug (gatifloxacin). In addition, barring *S. abortus equi*, for all other strains, the three compounds exhibited lesser potential as compared gatifloxacin. The reason for such results may due to the reduced binding to SipD orthologues.

**TABLE 2:** Inhibitory activity (MICs in μg/mL) of compounds 5, 19, and 32 against *Salmonella* sp.

Compd	*S. enteritidis*	*S. typhi*	*S. typhimurium*	*S. paratyphi*	*S. abortus equi*
5	31.6 ± 3.6	12.3 ± 2.1	8.0 ± 2.0	24.3 ± 3.5	5.0 ± 1.0
19	53.3 ± 4.9	31.6 ± 5.0	19.1 ± 3.1	16.3 ± 3.1	3.0 ± 1.0
32	22.0 ± 3.0	44.0 ± 4.6	34.3 ± 3.8	22.0 ± 2.6	1.2 ± 0.3
Gatifloxacin	7.7 ± 1.5	9.7 ± 2.5	3.3 ± 1.2	3.0 ± 1.7	2.0 ± 1.0

a The antibacterial tests were carried out three times, and the MICs, value was expressed as mean ± SD.

### *In vitro* Cytotoxicity of Compounds 5, 19, and 32

**FIGURE 5:**
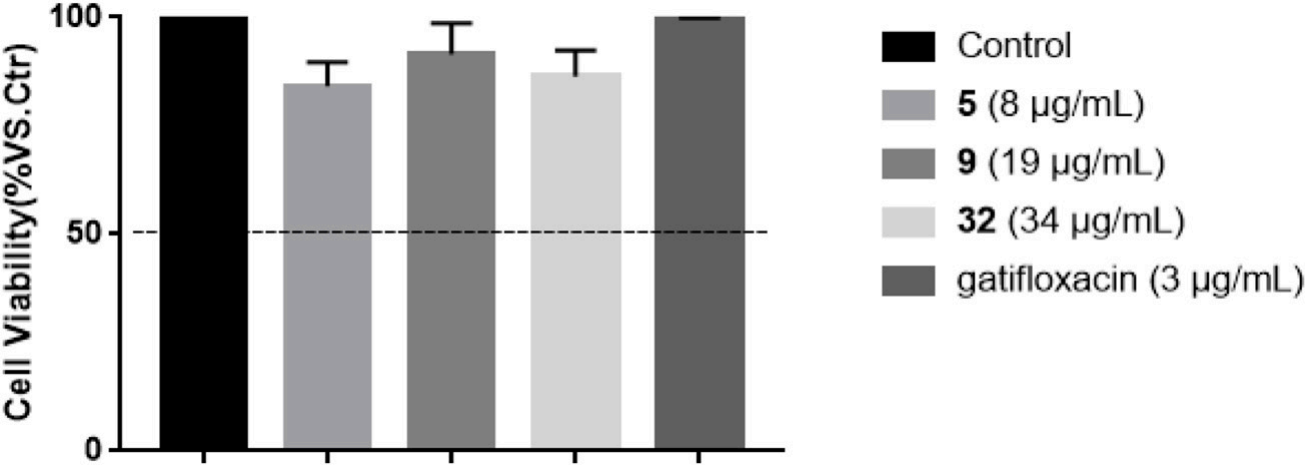
Effects of compounds 5, 19, 32, and gatifloxacin on the cell viability of RAW 264.7 cells for 24 h. Cell viability was expressed as percent cell viability compared to that of DMSO vehicle control cells (100%), and cell viability more than 50% at their respective concentration was considered to be non-toxic.

The cytotoxicity of compounds 5, 19, 32 against RAW 264.7 cells at the concentrations based their respective MICs towards *S. typhimurium* was initially evaluated using MTT assay (Zhang et al., 2020). The results in Figure 5 showed that none of these compounds was toxic towards RAW 264.7 cells, with the cell viability of 84.24 ± 5.45% (5, 8 μg/ml), 91.50 ± 7.09% (9, 19 μg/ml), and 86.36 ± 6.04% (32, 34 μg/ml)), respectively, compared with untreated cell group.

### Intracellular Killing Assay of Compounds 5, 19, and 32

**FIGURE 6:**
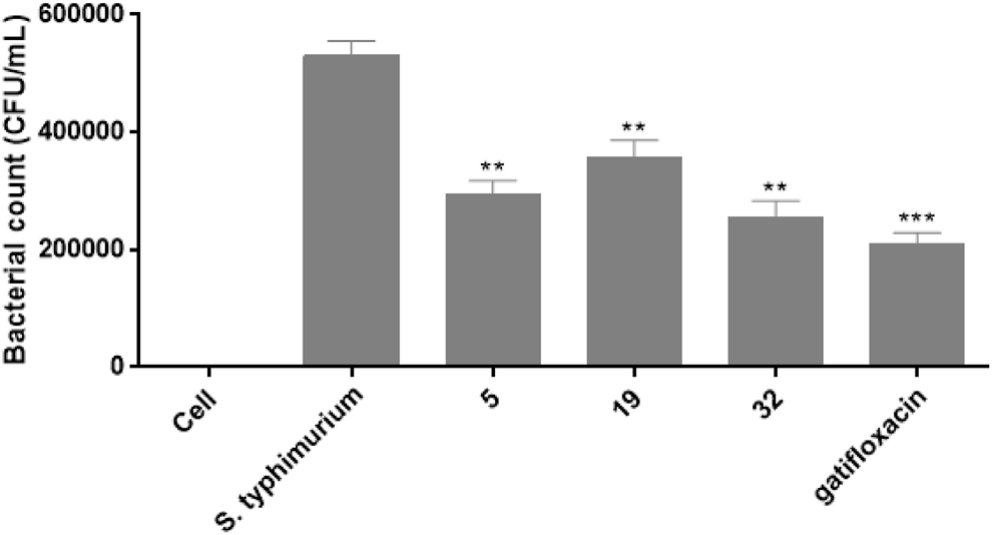
The effects of compounds 5, 19, 32, and gatifloxacin on the survival of *S. typhimurium* in RAW 264.7 cells.

The intracellular anti*-Salmonella activity* of compounds 5, 19, and 32 was then evaluated in the model of RAW 264.7 cells infected with *S. typhimurium* according to the protected protocol (Birhanu et al., 2018). The concentrations of these tested compounds were selected on the basis of their MIC values. Based on the results of intracellular killing assay shown in Figure 6, All these compounds 5, 19, and 32 could decrease the intracellular-survival of *S. typhimurium* by 44.4, 32.5, and 52.2%, respectively, compared with the non-treated *S. typhimurium* group. The intracellular survival suppression by the positive control gatifloxacin was 60.2%.

### Prediction of ADMET Properties

The ADMET profiling for the identified three compounds were predicted. From the results (Table 3), we can see that all of the three compounds were predicted to have acceptable solubility and good absorption level, and displayed moderate BBB penetration level. Importantly, both of them may not bind to PPB and CYP2D6, which possibly indicated fewer side effects of these three compounds.

**TABLE 3:** Prediction of ADMET properties of the identified compounds.

No	PSA-2D	AlogP98	Solubility level	Absorption level	BBB level	PPB	CYP2D6	Hepatotoxic prediction
5	77.90	2.928	2	0	2	0.66499	False	True
19	113.04	0.691	3	0	4	0.62488	False	True
32	77.75	1.773	3	0	3	5.81491	False	True

PSA-2D: polar surface area; AlogP98: Lipophilicity descriptor; Solubility Level: (0, Good; 1, Moderate; 2, Poor; 3, Very poor); Absorption Level: (0, Good; 1, Moderate; 2, Poor; 3, Very poor); BBB, Level: (0, very high blood–brain barrier penetration; 1, high; 2, medium; 3, low; 4, undefined); PPB: plasma protein binding; CYP2D6: Cytochrome P450 2D6 inhibition.

## Conclusion

In this study, three novel T3SS inhibitors 5, 19, and 32 with different structural scaffold were first discovered based on virtual screening, and the *in vitro* anti-bacterial activities of these inhibitors against five stains of *Salmonella* sp. were evaluated. Finally, the activities of these compounds against a panel of *Salmonella* bacteria were tested in anti-bacterial bioassay, and the results showed all of them exhibited promising anti-*Salmonella* activity with MICs values ranging from 1 to 53 μg/ml. Further experiment revealed intracellular-inhibition of these compounds against *S. typhimurium*. It was worth to note that all these three inhibitors showed potent activity towards *S. abortus equi* as that of gatifloxacin. Based on these results, we first confirmed that the T3SS tip protein SipD is a potential target for T3SS inhibitor discovery, and these new T3SS inhibitors 5, 19, and 32 could be lead compounds used in the discovery of drugs against the infection of *Salmonella* bacteria, especially *S. abortus equi*, and their further structural modification and bioactive optimization are deserved. And when we performed structural modification, we would first predict the drug-likeness properties of the designed compounds.

## Materials and Methods

### Molecular Docking Based Virtual Screening

Molecular docking was performed using the Autodock 4.2 program (; Morris et al., 1998; Huey et al., 2007). The crystal structure of the *Salmonella* type III secretion system tip protein SipD in complex with deoxycholate (PDB ID: 3O01) (Chatterjee et al., 2011) was used to construct the docking model. The missing hydrogen atoms were added, Gasteiger charges was assigned, and the protein were parameterized with AD4 type by Autodock Tools 1.5.6. Finally, the protein structure was used as an input for the Autogrid program. Grid map with 60 × 60 × 60 points was made according to the conformation of ligand, and the grid spacing was set to 0.375 Å. Rigid ligand docking was performed for prepared SPECS database compounds. Docking calculations were carried out using the Lamarckian genetic algorithm (LGA). “Clustering Molecules” protocols embedded in Pipeline Pilot 7.5 (Pipeline Pilot; Accelrys Software Inc., San Diego, CA) was used to do the cluster analysis. Finally, according to the cluster analysis results, the candidate compounds were selected and purchased from SPECS database supplier.

### Screening of Small Molecules for Inhibition of *Salmonella* Invasion

The effect of T3SS inhibitor candidates on the ability of *S. typhimurium* to invade a cultured human epithelial cell line (Henle 407) was performed in a method similar to one previously described (Chatterjee et al., 2011). Henle 407 cells were grown in DMEM with 10% fetal calf serum at 37°C in 5% CO_2_ in 24-well plates. The plasmid pRK2-SipD with WT SipD was electroporated into the *S. typhimurium SipD* ^−^ strain and single colonies were inoculated into LB media supplemented with 25 mg/L trimethoprim, 50 mg/L ampicillin, 50 mg/L kanamycin and grown in standing cultures overnight. A 10 ml LB culture with 1 mM IPTG was inoculated with 1 ml of overnight bacterial growth, and incubated at 37°C on standing for 2.5–3 h. Approximately 15–30 uL of bacterial suspension was added with 300 uL DMEM into the Henle 407 cells and incubated at 37°C for 60 min to allow invasion before the suspension was removed by aspiration. The Henle 407 cells were incubated with fresh DMEM with 100 mg/L of gentamycin for 1.5 h, aspirated, rinsed with DMEM, and lysed with 1% Triton X-100 to free the entrapped bacteria. The number of bacterial colonies, which correlated with invasiveness, was estimated by serial dilution and plating. The *Salmonella* invasion assay was done in triplicate.

### *In vitro* anti-Salmonella Bioassay

The anti-*Salmonella* activities of these compounds were performed according to the previous reported protocol (Wei et al., 2016), using the minimum inhibitory concentration (MIC) with different strains, including *S. enteritidis*, *S. typhi*, *S. typhimurium*, *S. paratyphi*, *S.* and *abortus equi*. Gatifloxacin was used as positive controls. The test compounds 5, 19, and 32 in DMSO were prepared and then poured into 96-well plates. The final concentration of o.39–100 μg/ml underwent a twofold serial dilution. The bacteria were incubated with a series of different concentrations of compounds at 37°C for 24 h. The microbacterial growth was measured at the absorption of 630 nm. All experiments were carried out in triplicate.

### *In vitro* Cytotoxicity Assay

To study the cytotoxic effects of compounds on cell viability, the RAW 264.7 cells were seeded into 96-well plates at 1 ×10^4^ cells/well and allowed to attach for 24 h. The medium was replaced with 100 μL medium containing the indicated concentrations of compounds and further incubated for 24 h. Each well was added 10 μL MTT (5 mg/ml in PBS) and the plates were incubated for 4 h at 37°C. Supernatants were aspirated and formed formazan was dissolved in 100 μL of dimethyl sulfoxide (DMSO). The optical density (OD) was measured at an absorbance wavelength of 490 nm using a Microplate Reader (Tecan, Switzerland).

### Intracellular Killing Assay

The intracellular killing experiment was performed according to the previous reported protocol (Birhanu et al., 2018). RAW 264.7 cells (10^5^ cells/ml) were cultured in 24-well plates, and then treated with *S. Typhimurium* (10^7^ CFU/ml) and further incubated for 45 min. After the cells were washed, the compound 5 (8 μg/ml), 9 (19 μg/ml) and 32 (34 μg/ml) or gatifloxacin (3 μg/ml) were respectively added and incubated for 1 h at 37 °C. Finally, cells were treated with gentamicin (100 μg/ml) for 1 h and lysed with 0.1% of trition × 100 before being serially diluted and plated on LB agar. The cells infected with *S. Typhimurium* without treatment was used as the control.

### Statistical Analysis

All data are presented as the mean ± standard deviation. Data were processed using 17.0 SPSS software (SPSS Inc., Chicago, IL, United States ). Statistical comparisons were analyzed using one-way analysis of variance (ANOVA). *p* values of less than 0.05 were considered to be statistically significant. *p < 0.05, **p < 0.01, and ***p < 0.001.

## Data Availability

The raw data supporting the conclusions of this article will be made available by the authors, without undue reservation.
