# Supplementary material for: Identification of Novel Type Three Secretion System (T3SS) Inhibitors by Computational Methods and Anti-Salmonella Evaluations
Source: Front Pharmacol. 2021 Nov 16;12:764191. doi: 10.3389/fphar.2021.764191 (PMC8635108; doi:10.3389/fphar.2021.764191)
Supplement: Supplementary file 1 [file DataSheet1.docx]

Supplementary data for

Identification of novel Type three secretion system (T3SS) inhibitors by computational methods and anti-*Salmonella* evaluations

Yonghui Wang,^1^ Meihui Hou,^2^ Zhaodong Kan^3^, Guanghui Zhang^3^, Yunxia Li^3^, Lei Zhou^4,^* and Changfa Wang^1,^*

^1^College of Agronomy, Liaocheng University, Liaocheng 252059, China

^2^Burns and plastic surgery department, The 960th Hospital of the PLA Joint Logistics Support Force, Jinan 252059, China

^3^Laizhou city laiyu chemical co., Ltd, Laizhou 261400, China

^4^School of Biological Science and Technology, University of Jinan, Jinan 250022, China

*** Correspondence:**Lei Zhou
[bio_zhoul@ujn.edu.cn](mailto:bio_zhoul@ujn.edu.cn)
Changfa Wang
[wangcf1967@163.com](mailto:wangcf1967@163.com)


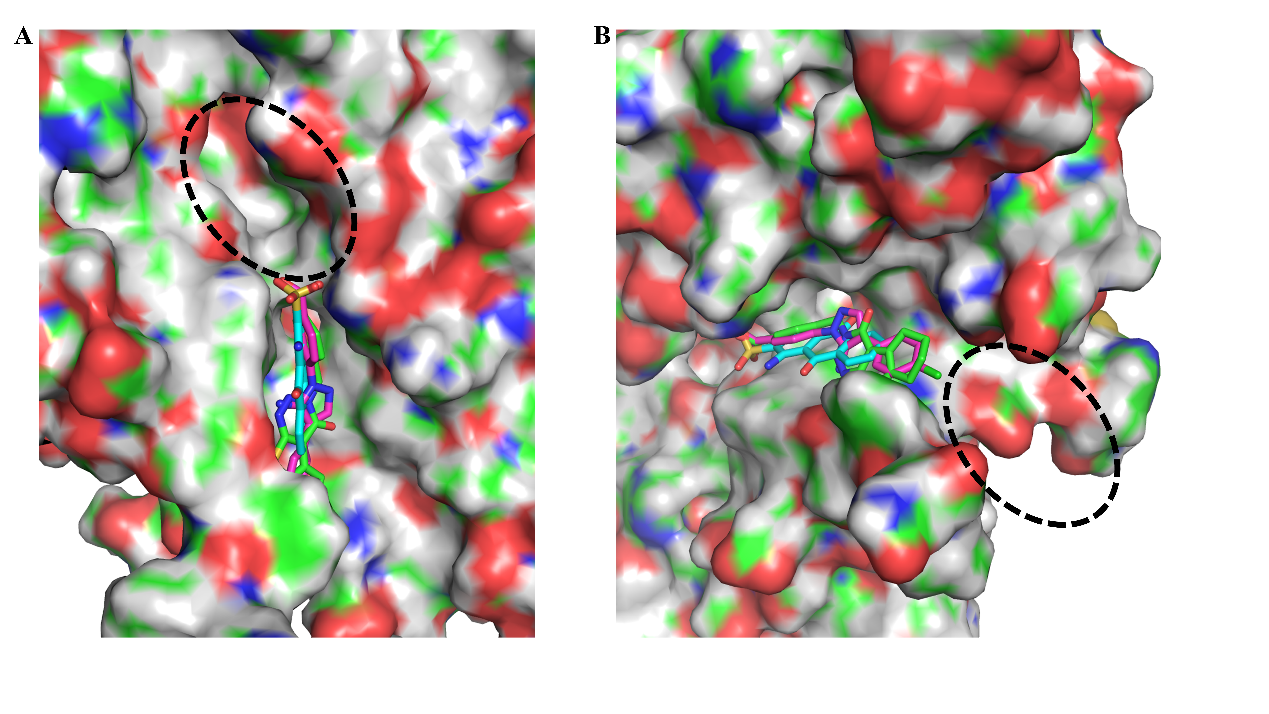


Figure 1. The predicted binding modes of compounds 5, 19, and 32 with SipD protein.

**Virtual screening method:**

Molecular docking based virtual screening was performed using the Autodock 4.2 program (Huey et al., 2007; Morris et al., 1998). The crystal structure of the *Salmonella* type III secretion system tip protein SipD in complex with deoxycholate (PDB ID: 3O01) (Chatterjee et al., 2011) was used to construct the docking model. The missing hydrogen atoms were added, Gasteiger charges was assigned, and the protein were parameterized with AD4 type by Autodock Tools 1.5.6. Finally, the protein structure was used as an input for the Autogrid program. Grid map with 60 x 60 x 60 points was made according to the conformation of ligand, and the grid spacing was set to 0.375 Å. Rigid ligand docking was performed for prepared SPECS database ([www.specs.com](http://www.specs.com), contains more than 200,000 compounds) compounds. Docking calculations were carried out using the Lamarckian genetic algorithm (LGA). After molecular docking simulation, the top 300 compounds were selected for the following cluster analysis. “Clustering Molecules” protocols embedded in Pipeline Pilot 7.5 (Pipeline Pilot; Accelrys Software Inc., San Diego, CA) was used to do the cluster analysis. Finally, according to the cluster analysis results, the 46 candidate compounds were selected and purchased from SPECS database supplier.
